# A critical role for CK2 in cytokine-induced activation of NFκB in pancreatic β cell death

**DOI:** 10.1007/s12020-013-0133-6

**Published:** 2013-12-24

**Authors:** Caroline Jaksch, Peter Thams

**Affiliations:** Department of Biomedical Sciences, University of Copenhagen, 3C Blegdamsvej, 2200 Copenhagen N, Denmark

**Keywords:** β cell, Islet, CK2, Cytokine, NFκB, STAT1

## Abstract

This study aimed to assess the role of constitutive protein kinase CK2 in cytokine-induced activation of NFκB in pancreatic β cell death. The CK2 inhibitors DRB (5,6-dichloro-1-β-d-ribofuranosylbenzimidazole) (50 μM) and DMAT (2-dimethylamino-4,5,6,7-tetrabromo-1*H*-benzimidazole) (5 μM), which decreased CK2 activity by approx. 65 %, rescued INS-1E β cells and mouse islets from cytokine (IL-1β, TNF-α plus IFN-γ)-induced β cell death without affecting H_2_O_2_- or palmitate-induced β cell death. Western blot analysis revealed that while DRB or DMAT did not influence cytokine-induced IκBα degradation, they inhibited NFκB-dependent IκBα resynthesis, demonstrating that cytokine-induced NFκB activity is dependent on CK2. Both DRB and DMAT inhibited the constitutive phosphorylation of NFκB p65 at serine 529, while leaving cytokine-induced phosphorylations of NFκB p65 at serines 276 and 536 unaltered. In comparison, putative phosphorylation sites for CK2 on HDACs 1, 2, and 3 at serines 421/423, 394, and 424, respectively, which may stimulate NFκB transcriptional activity, were unchanged by cytokines and CK2 inhibitors. Whereas IL-1β and TNF-α stimulate IκBα degradation and NFκB activation, IFN-γ potentiates cytokine-induced β cell death through activation of STAT1. DRB and DMAT inhibited IFN-γ-stimulated phosphorylation of STAT1 at serine 727, while leaving IFN-γ-induced phosphorylation of STAT1 at tyrosine 701 unaffected. Inhibition of cytokine-induced β cell death by CK2 inhibitors was, however, not dependent on IFN-γ, and IFN-γ did not affect CK2-dependent IκBα turnover. In conclusion, it is suggested that cytokine-induced activation of NFκB in β cells is dependent on CK2 activity, which phosphorylates NFκB p65 at serine 529.

## Introduction

Diabetes mellitus is characterized by a decline in β cell mass and function leading to insufficient insulin secretion and hyperglycemia [1]. In type 1 diabetes mellitus, pancreatic islet inflammation contributes to the progressive loss of insulin producing β cells and inflammatory mediators, e.g., IL-1β, TNF-α, and IFN-γ contribute to the suppression of β cell function and subsequent apoptosis [2, 3]. In addition, recent evidence suggests that islet inflammation may contribute to pancreatic β cell death in type 2 diabetes mellitus [4].

CK2 (formerly casein kinase 2) is a highly conserved serine/threonine kinase [5], which also has been identified in β cells [6]. This constitutively active and ubiquitously expressed enzyme is known to phosphorylate more than 300 substrates and controls a wide range of processes, including the regulation of cell cycle, apoptosis, and inflammation. Although CK2 is a constitutive active enzyme, its activity has been described to be modulated by several factors, including IL-1β, TNF-α, and IFN-γ, which have been shown to stimulate CK2 activity in different cell types [5]. Moreover, CK2 phosphorylates and stimulates several cytokine-stimulated transcription factors, including nuclear factor-kappa B (NFκB) and signal transducer and activator of transcription (STAT1), key regulators of the inflammatory response [5].

NFκB is activated by a range of stimuli, including inflammatory cytokines, like IL-1β and TNF-α, and is generally regarded as one of the most important master regulators of the inflammatory response [7]. NFκB is a dimer composed of the five mammalian Rel proteins, p65-RelA, c-Rel, RelB, NFκB1-p50, and NFκB2-p52 where p65–p50 is the predominant form in β cells [2]. In unstimulated cells, NFκB dimer is complexed with proteins called inhibitor of κB (IκBs), which sequester the transcription factor in the cytoplasm by masking their nuclear localization sequence. Activators of NFκB initiate multiple intracellular signaling pathways that converge at IκB kinase (IKK) complex, which phosphorylates IκBs. This phosphorylation is a prerequisite for the subsequent ubiquitinylation and degradation of IκBs via the proteasomal pathway. NFκB is now free to migrate into the nucleus and activate gene transcription by binding to its recognition sequence in the promoter and enhancer regions of target genes [7].

CK2 has been found to phosphorylate IκBα at a number of sites, including serines 283, 289, 293, and threonine 291, and this has been found, at least in certain context, to trigger the degradation of the protein and thereby activation of NFκB [8, 9]. In addition to IκB, the NFκB p65 subunit has also been found to be subject to CK2-mediated phosphorylation with implications to inflammation [10]. NFκB p65 is phosphorylated at serines 276, 529, and 536, and whereas CK2 may increase phosphorylation of serine 276 and 536, serine 529 has been described to be a specific substrate for CK2 [11–14].

In addition, NFκB activity in β cells is dependent on histone deacetylases (HDACs), which through deacetylation of histones promote NFκB binding to DNA and gene expression [15, 16]. HDAC1, HDAC2, and HDAC3, which are present in β cells, have been shown to be phosphorylated by CK2 at serines 421/423, serine 394, and serine 424, respectively [17–19]. Overall, therefore, these findings suggest a potentially important role for CK2 in the regulation and expression of genes implicated in inflammation through the control of NFκB action.

In most tissues, NFκB activation inhibits cell death. However, under certain circumstances, or in certain cell types as in β cells, activation of NFκB may promote cell death [2]. IL-1β mediates activation of the transcription factor NFκB pathway, a pathway partly shared by TNF-α. IFN-γ signaling involves induction of JAKs 1 and 2, leading to activation of STAT1 through phosphorylation of STAT1 at tyrosine 701 [2]. In addition, IFN-γ action may be fortified by a CK2-dependent phosphorylation of STAT1 at serine 727 [20]. The concerted action of activated transcription factors initiates a cascade of events leading to the deleterious effects on β cells and to apoptosis in type 1 diabetes mellitus. In this process, NFκB appears to play a central role. Thus, whereas IL-1β and TNF-α trigger NFκB activation, induction of iNOS and apoptosis, IFN-γ fortifies β cell death by mechanisms which may involve potentiation of NFκB activity, and iNOS expression [3, 21]. Overall, therefore, CK2 may be speculated to promote cytokine-induced NFκB activity and β cell death through phosphorylations of IκBα, NFκB p65, STAT1, and HDACs 1, 2, and 3.

While the concept of *insulitis* in type 1 diabetes mellitus is well-established, its role in type 2 diabetes mellitus is disputed [3]. Elevation of circulating nutrients such as glucose and free fatty acids (FFAs) induces an inflammatory response within numerous tissues in the body and has been shown to induce IL-1β release in human and rodent islets [4]. Glucolipotoxicity in type 2 diabetes mellitus leading to β cell death has, however, been described to proceed independent of NFκB activation [3, 22, 23] and may rely on endoplasmic reticulum stress in response to prolonged elevation in FFAs [3, 22]. Furthermore, prolonged elevations in glucose and FFAs may lead to oxidative stress with formation of reactive oxygen species (ROS) like O _2_^•−^, OH^•^, and H_2_O_2_, which may stimulate β cell death through NFκB-independent pathways [3, 23].

The role of CK2 in β cell inflammation and apoptosis has not been investigated. This study, therefore, aimed at investigating the role of CK2 in cytokine-, FFA-, and ROS-induced β cell death. It is demonstrated that constitutive active CK2 does not affect palmitate- or H_2_O_2_-induced β cell death. CK2, however, stimulates cytokine-induced NFκB activity, most likely through phosphorylation of NFκB p65 at serine 529, and furthermore, phosphorylation of STAT1 at serine 727 appears to be dependent on CK2. Thus, in comparison with numerous other cell types, CK2 seems to have a selective pro-apoptotic function in pancreatic β cells.

## Materials and methods

### Materials

Crude bacterial collagenase was obtained from Boehringer (Mannheim, Germany). DRB (5,6-dichloro-1-β-d-ribofuranosylbenzimidazole), DMAT (2-dimethylamino-4,5,6,7-tetrabromo-1*H*-benzimidazole), and andrographolide were from Calbiochem (San Diego, CA). Recombinant mouse IL-1β was from PharMingen/BD Bioscience (San Jose, CA), recombinant rat IFN-γ and recombinant rat TNF-α were from R&D Systems (Abingdon, Oxon, UK). Antibodies for IκBα, NFκB p65, pNFκB p65 (ser 276), pNFκB p65 (ser 529), pNFκB p65 (ser 536), and pHDAC2 (ser 394) were from Santa Cruz Biotechnology, Inc. (Dallas, Texas). Antibodies for β-actin, pHDAC3 (ser 424), pSTAT1 (ser 727), and pSTAT1 (tyr 701) were from Cell Signalling Technology (Danvers, MA). Antibody for pHDAC1 (ser 421/ser 423) was from Millipore (Billerica, MA). All other chemicals were of analytical grade. INS-1E cells were kindly provided by Claes Wollheim, Geneva.

### Isolation and culture of islets

Islets were prepared by collagenase digestion of the pancreas of male albino mice (NMRI) (approx. 18–22 g body weight) (Charles River Laboratories Sulzfeld, Germany) fed ad libitum on a standard laboratory diet. Principles of laboratory animal care as approved by The Danish Animal Experiments Inspectorate were followed. Islets were kept in tissue culture for 48–72 h in a 5 % CO_2_ incubator in RPMI 1640 medium (11 mM glucose) supplemented with glutamax (2 mM), 10 % (v/v) newborn calf serum (Gibco, Paisley, Strathclyde, UK), 100 units penicillin/ml, and 100 μg streptomycin/ml. Cytokines [IL-1β (160 pg/ml), TNF-α (2 ng/ml), and IFN-γ (200 pg/ml)] and other test substances as specified in the text were present during the last 48 h of culture.

### Culture of INS-1E cells

INS-1E cells were cultured in 12-well plates (2.56 × 10^5^ cells/well) or 3 cm dishes (1.25 × 10^6^ cells/dish) in a 5 % CO_2_ incubator in RPMI 1640 medium (11 mM glucose) supplemented with glutamax, 10 % (v/v) fetal calf serum (Gibco, Paisley, Strathclyde, UK), 100 units penicillin/ml, 100 μg streptomycin/ml, and 50 μM mercaptoethanol. After 48 h of culture, the serum concentration was lowered to 0.5 % fetal calf serum for the remaining culture period with addition of cytokines [IL-1β (80 pg/ml), TNF-α (1 ng/ml), and IFN-γ (100 pg/ml)] and other test substances as specified in the text. This low concentration of serum did not affect cell viability [24].

### MTT assay

The proportion of viable cells in treated versus control cells was determined using the MTT [3-(4,5-dimethylthiazol-2-yl)-2,5-diphenyltetrazolium bromide] assay, which measures succinate dehydrogenase activity [25]. Briefly, INS-1E cells or groups of 50 islets were incubated with 500 μg MTT/ml RPMI 1640 medium for 3 h at 37 °C. At the end of the incubation, the medium containing MTT was removed, and islets or cells were dissolved in DMSO. Absorbance was then measured at 540 and 690 nm [24].

### Apoptosis assay

The proportion of apoptotic cells in treated versus control islets was determined by the Cell Death Detection ELISA^PLUS^ kit (Roche Diagnostics, Mannheim, Germany). Briefly, groups of 25 islets were lysed in 200 μl lysis buffer (Roche Diagnostics, Mannheim, Germany) followed by measurement of histone-associated DNA fragments as outlined by the manufacturer.

### Western blot analysis

After culture, INS-1E cells were washed twice in PBS++, lysed in 100 μl radioimmunoprecipitation assay (RIPA) buffer (1 % Triton X-100, 0.5 % sodium deoxycholate, 0.1 % sodium dodecyl sulfate, 1 mM 4-(2-aminoethyl)benzene sulfonyl fluoride, 1 mM orthovanadate, 2 μM okadaic acid, 10 mM β-glycerophosphate, 10 mM NaF, 1 μg/ml aprotinin, and 1 μg/ml leupeptin in PBS), and centrifuged at 11,000×*g* for 3 min. Protein concentration was then determined in supernatants using the BCA protein assay (Sigma Chemical Co., St. Louis, MO). 50 μg of protein was loaded and separated by electrophoresis on 7 % NuPage Novex Tris-Acetate gels in Tris-Acetate running buffer or 12 % NuPage Novex Bis–Tris gels in Mops running buffer as appropriate (Invitrogen, Taastrup, Denmark) and transferred to nitrocellulose membranes by electroblotting. After incubation of the membrane in blocking buffer (Tris-buffered saline containing 5 % nonfat dry milk), the membrane was exposed overnight at 4 °C to primary antibody diluted 1:1,000 in blocking buffer. The membrane was subsequently washed in Tris-buffered saline containing 0.1 % Tween 20, incubated with peroxidase-conjugated goat antirabbit IgG (Dako, Glostrup, Denmark), and proteins detected by chemiluminescence using ECL plus Western blotting detection reagent (GE Healthcare, Chalfont St. Giles, UK). Proteins were then quantified relative to controls on the same gel with ImageJ software as described [26]. β-actin served as a loading control.

### CK2 assay

After culture, INS-1E cells or mouse islets were washed in PBS, lysed in 100 μl lysis buffer (1 % Triton X-100, 1 mM 4-(2-aminoethyl)benzene sulfonyl fluoride, 1 mM orthovanadate, 2 μM okadaic acid, 10 mM β-glycerophosphate, 10 mM NaF, 1 μg/ml aprotinin, 1 μg/ml leupeptin, and 5 mM EGTA in 20 mM Mops, pH 7.2), and centrifuged at 11,000×*g* for 3 min. Protein concentration was then determined in supernatants using the BCA protein assay (Sigma Chemical Co., St. Louis, MO). CK2 activity was measured in a phosphotransferase assay with 10 μM [γ-^32^P] ATP (Perkin-Elmer, Skovlunde, Denmark) as phosphate donor and 200 μM RRRDDDSDDD as phosphate acceptor as outlined by the manufacturer (Millipore, Billerica, MA).

### Insulin secretion

Insulin release from islets was determined by perifusion in a noncirculating system with beads of 0.25 ml Bio-Gel P2 (Bio-Rad Laboratories, Rockville Center, NY, USA) as a supporting medium. 25 islets per chamber were perifused at 37 °C at a flow rate of 0.26 ml/min. The perifusion medium was Krebs–Ringer medium supplemented with 20 mM HEPES, 5 mM NaHCO_3_, 2 mg/ml of bovine serum albumin/ml, and 3.3 mM glucose. Islets were initially perifused for 45 min to obtain a basal release rate and then stimulated with 16.7 mM glucose for 60 min. The effluent medium was collected for periods of 5 or 10 min. Insulin was determined by radioimmunoassay [27].

### Islet insulin content

Groups of ten islets were collected in 1 ml of acidified ethanol (0.7 M HCl/ethanol, 1:3 v/v) and incubated for approx. 20 h at 4 °C for extraction of insulin [28].

### Miscellaneous

DRB, DMAT, and andrographolide were added in a small volume of DMSO, final conc. 0.01–0.1 %. Results are given as mean ± SEM for *n* experiments. Statistical evaluation of the data was made by *t* test or ANOVA, followed by the Newman–Keuls test for multiple comparisons; not significant, *P* > 0.05.

## Results

### DRB and DMAT inhibit cytokine-induced β cell death

**Fig. 1:**
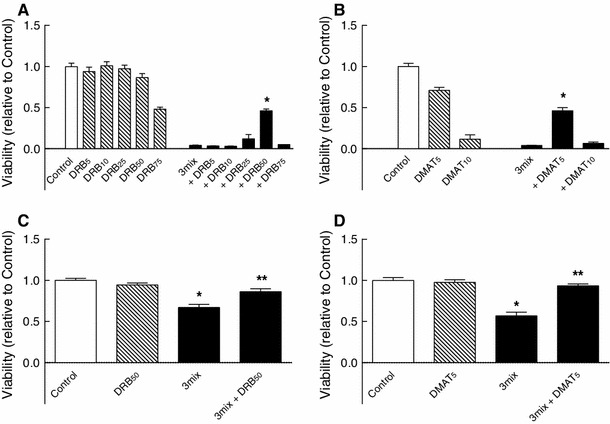
Effects of DRB and DMAT on β cell survival. **a**, **b** INS-1E cells and **c**, **d** mouse islets were cultured for 48 h in the absence or presence of cytokines (IL-1β, TNF-α, and IFN-γ) (3mix) and with DRB (5–75 μM) (DRB_5–75_) or DMAT (5–10 μM) (DMAT_5–10_) as indicated before determination of cell viability. **a**, **b** Results are mean ± SEM (*n* = 4–12); **a** *P < 0.001 versus 3mix; **b** *P < 0.001 versus 3mix. **c**, **d** Results are mean ± SEM (*n* = 4); **c** *P < 0.001 versus control, **P < 0.001 versus 3mix; **d** *P < 0.001 versus control, **P < 0.001 versus 3mix

To evaluate the role of CK2 in β cell survival, INS-1E cells were exposed to increasing concentrations of the CK2 inhibitor DRB (Fig. 1a). While DRB in concentrations above 50 μM appeared detrimental to INS-1E cells, DRB at 50 μM and below did not affect β-cell survival. 50 μM of DRB, however, led to a significant protection against β cell death induced by a cytokine mixture of IL-1β, TNF-α, and IFN-γ. In addition, DMAT, another CK2 inhibitor, at a concentration of 5 μM, with minimal effect on β cell survival per se, also increased β-cell survival during a 48 h exposure to cytokines (Fig. 1b).

**Fig. 2:**
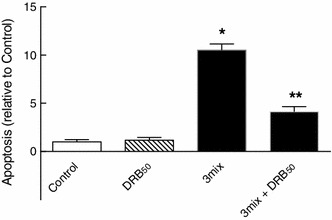
Role of CK2 in cytokine-induced β cell apoptosis. Mouse islets were cultured for 48 h in the absence or presence of cytokines (IL-1β, TNF-α, and IFN-γ) (3mix) and with DRB (50 μM) (DRB_50_) as indicated before determination of apoptosis. Results are mean ± SEM (*n* = 4), *P < 0.001 versus control, **P < 0.001 versus 3mix

Furthermore, both DRB (50 μM) and DMAT (5 μM), which per se did not affect the survival of mouse islets, improved survival of mouse islets during a 48 h exposure to cytokines (Fig. 1c, d). In accordance, DRB (50 μM) also prevented cytokine-induced apoptosis in mouse islets (Fig. 2), suggesting that CK2 may promote cytokine-induced death of β cells.

**Fig. 3:**
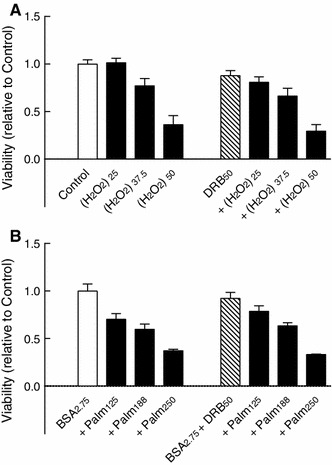
Role of CK2 in H_2_O_2_- and palmitate-induced β cell death. INS-1E cell were cultured for 24 h with **a** H_2_O_2_ (25–50 μM) [(H_2_O_2_)_25–50_] or **b** palmitate (125–250 μM) (Palm_125–250_) plus bovine serum albumin (2.75 mg/ml) (BSA_2.75_) and with DRB (50 μM) (DRB_50_) as indicated before determination of cell viability. Results are mean ± SEM (*n* = 4–6)

In comparison, DRB (50 μM) failed to affect both H_2_O_2_- and palmitate-induced demise of INS-1E β cells (Fig. 3), suggesting that CK2 may have a specific role in promoting cytokine-induced β cell death.

DRB (50 μM) and DMAT (5 μM) caused an approximately 65 % inhibition of CK2 activity, measured as phosphorylation of the CK2 substrate RRRDDDSDDD, in cell extracts of both INS-1E cells and mouse islets (Table 1).

**Table 1:** Effects of DRB and DMAT on CK2 activity

	CK2 activity (pmol/ng/min)			*P*
	Control	DRB (50 μM)	DMAT (5 μM)	
INS-1E β cells	10.45 ± 1.83 (3)	3.83 ± 0.40 (3)		<0.05
	11.16 ± 0.63 (3)		2.95 ± 0.11 (3)	<0.005
Mouse islets	12.06 ± 1.62 (4)	4.61 ± 0.78 (4)		<0.01
	13.96 ± 1.32 (5)		4.91 ± 1.12 (5)	<0.001

Results are mean ± SEM (*n*)

### Role of NFκB in CK2-stimulated β cell death

**Fig. 4:**
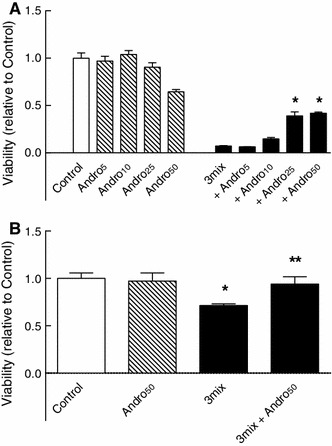
Role of NFκB in cytokine-induced β cell death. **a** INS-1E cells and **b** mouse islets were cultured for 48 h in the absence or presence of cytokines (IL-1β, TNF-α, and IFN-γ) (3mix) and with andrographolide (5–50 μM) (Andro_5–50_) as indicated before determination of cell viability. **a** Results are mean ± SEM (*n* = 8), *P < 0.001 versus 3mix, **b** Results are mean ± SEM (*n* = 4), *P < 0.05 versus control, **P < 0.05 versus 3mix

NFκB is a master regulator of cytokine-induced β-cell death and as demonstrated in Fig. 4 inhibition of NFκB by the NFκB inhibitor andrographolide protects both INS-1E cells and mouse islets from cytokine-induced β cell death. To investigate the potential role of NFκB in CK2-mediated β cell death, we, therefore, aimed at investigating the effect of CK2 inhibitors on NFκB activation and activity.

**Fig. 5:**
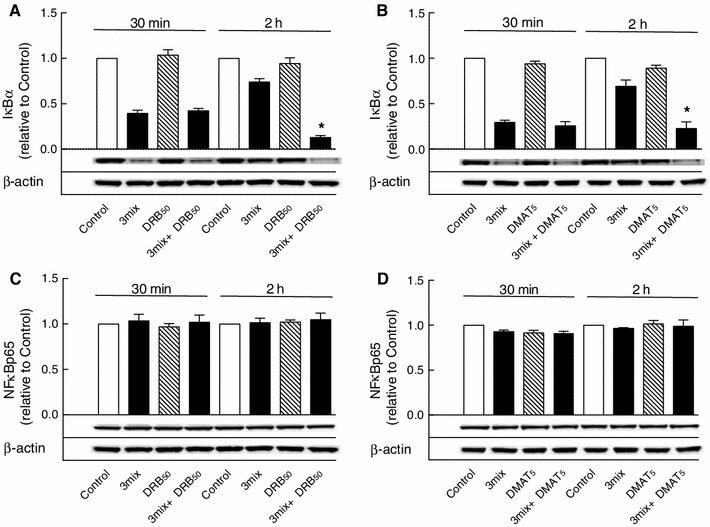
*Western blots* of IκBα and NFκB p65 proteins. INS-1E cells were cultured for 30 min or 2 h in the absence or presence of cytokines (IL-1β, TNF-α, and IFN-γ) (3mix) and DRB (50 μM) (DRB_50_) or DMAT (5 μM) (DMAT_5_) as indicated before determination of **a**, **b** IκBα and **c**, **d** NFκB p65 protein levels. Results are mean ± SEM (*n* = 3–6) with a representative experiment shown below *bars*. **a** *P < 0.001 versus 3mix; **b** *P < 0.001 versus 3mix

CK2 has been described to phosphorylate, and at least in certain context, to trigger the degradation of IκBα and thereby the activation of NFκB [8, 9]. However, neither DRB (50 μM) nor DMAT (5 μM) affected cytokine-induced degradation of IκBα as determined after 30 min of incubation (Fig. 5a, b).

IκBα contains a κB site in its promoter. To investigate whether CK2 affects NFκB transcriptional activity, the effects of DRB (50 μM) and DMAT (5 μM) on IκBα resynthesis were evaluated. As demonstrated in Fig. 5a, b, extended exposure of β cells to cytokines for 2 h led to a rapid resynthesis of IκBα, which was abrogated by both DRB (50 μM) and DMAT (5 μM), suggesting a critical role for CK2 in NFκB transcriptional activity.

In comparison, NFκB p65 expression as determined after 30 min and 2 h was not affected by CK2 inhibitors and cytokines (Fig. 5c, d).

In addition to IκBα, the NFκB p65 subunit has been found to be subject to CK2-mediated phosphorylation with implications to inflammation. Thus, while serine 529 has been identified as a specific phosphorylation site for CK2, other studies also point at serine 276 and serine 536 as possible targets for CK2 [11–14].

**Fig. 6:**
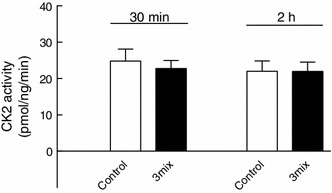
Effects of cytokines on CK2 activity in INS-1E cells. INS-1E cells were cultured for 30 min or 2 h in the absence or presence of cytokines (IL-1β, TNF-α, and IFN-γ) (3mix). Results are mean ± SEM (*n* = 6)

**Fig. 7:**
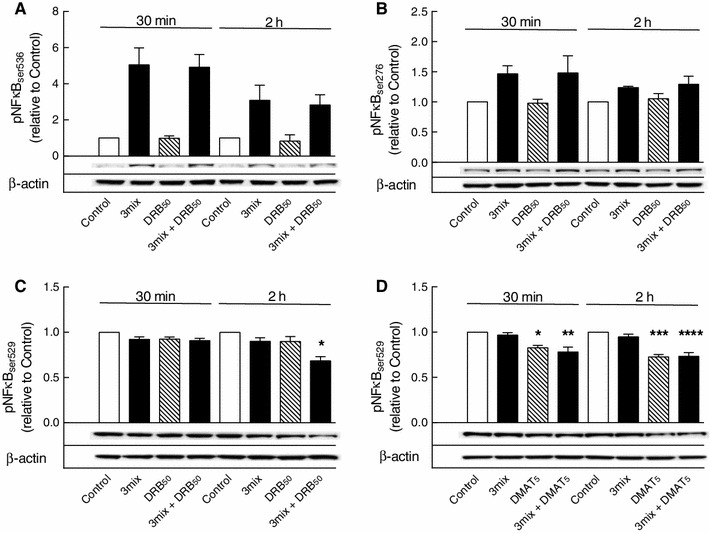
Effects of DRB and DMAT on NFκB p65 phosphorylations. INS-1E cells were cultured for 30 min or 2 h in the absence or presence of cytokines (IL-1β, TNF-α, and IFN-γ (3mix) and DRB (50 μM) (DRB_50_) or DMAT (5 μM) (DMAT_5_) as indicated. **a** NFκB p65 phosphoserine 536 (pNFκB_ser536_), **b** NFκB p65 phosphoserine 276 (pNFκB_ser276_), **c**, **d** NFκB p65 phosphoserine 529 (pNFκB_ser529_). Results are mean ± SEM (*n* = 3–7) with a representative experiment shown below *bars*. **c** *P < 0.001 versus control; **d** *P < 0.01 versus control, **P < 0.01 versus 3mix, ***P < 0.001 versus control, ****P < 0.001 versus 3mix

Culture of INS-1E cells for 30 min or 2 h with cytokines did not affect CK2 activity in cell extracts (Fig. 6), suggesting that cytokines may not increase CK2 activity in β cells. In accordance, exposure of INS-1E cells to cytokines led to phosphorylation of NFκB p65 at serine 536 and to a lesser extent at serine 276, which was insensitive to DRB (50 μM) (Fig. 7a, b).

In comparison, however, serine 529 appeared to be constitutively phosphorylated, and sensitive to dephosphorylation by both DRB (50 μM) (Fig. 7c) and DMAT (5 μM) (Fig. 7d), suggesting that constitutive CK2 activity may be necessary for NFκB activity.

### Role of HDACs 1, 2, and 3 in CK2-stimulated β cell death

**Fig. 8:**
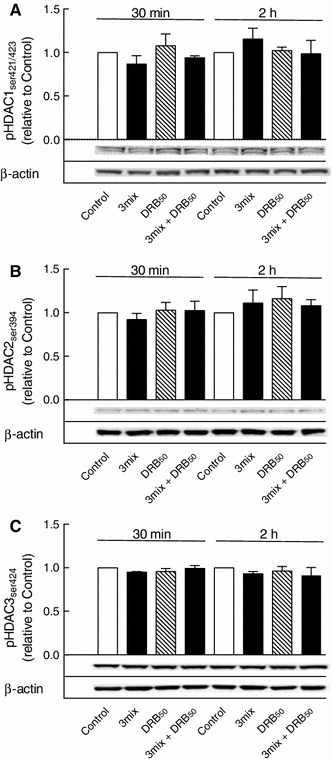
Effects of DRB on HDACs 1–3 phosphorylations. INS-1E cells were cultured for 30 min or 2 h in the absence or presence of cytokines (IL-1β, TNF-α, and IFN-γ) (3mix) and DRB (50 μM) (DRB_50_) as indicated. **a** HDAC1 phosphoserine 421/423 (pHDAC1_ser421/423_), **b** HDAC2 phosphoserine 394 (pHDAC2_ser394_), **c** HDAC3 phosphoserine 424 (pHDAC3_ser424_). Results are mean ± SEM (*n* = 3–5) with a representative experiment shown below *bars*

While NFκB phosphorylation may be important, NFκB transcriptional activity is also dependent on HDACs, which stimulate histone binding and NFκB transcriptional activity [15]. HDAC1, HDAC2, and HDAC3, which have been identified in β-cells [15, 16], have been described to contain CK2-sensitive phosphorylation sites at serines 421/423, serine 394, and serine 424, respectively [17–19]. DRB (50 μM) did not, however, affect phosphorylation of either HDAC1 at serine 421/423, HDAC2 at serine 394 or HDAC3 at serine 424, suggesting that these sites may not determine CK2-dependent NFκB activity (Fig. 8).

### Role of STAT1 in CK2-stimulated β cell death

Inhibition of CK2 has been described to attenuate the IFN-γ-regulated expression of several key genes in other tissues, and this is mediated, at least in part, via modulation of STAT1 phosphorylation on serine 727, necessary for maximal activity [20].

**Fig. 9:**
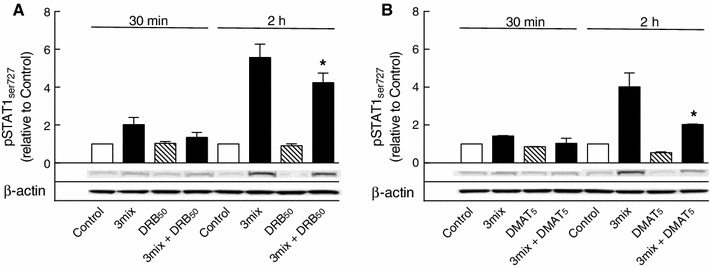
Effects of DRB and DMAT on STAT1 serine 727 phosphorylation. INS-1E cells were cultured for 30 min or 2 h in the absence or presence of cytokines (IL-1β, TNF-α, and IFN-γ) (3mix) and DRB (50 μM) (DRB_50_) or DMAT (5 μM) (DMAT_5_) as indicated before determination of STAT1 phosphoserine 727 (pSTAT1_ser727_). Results are mean ± SEM (*n* = 3–5) with a representative experiment shown below *bars*. **a** *P < 0.01 versus 3mix; **b** *P < 0.001 versus 3mix

In accordance DRB (50 μM) and DMAT (5 μM) inhibited cytokine-induced phosphorylation of STAT1 at serine 727, suggesting a potential significance of STAT1 in CK2-dependent β-cell death (Fig. 9).

**Fig. 10:**
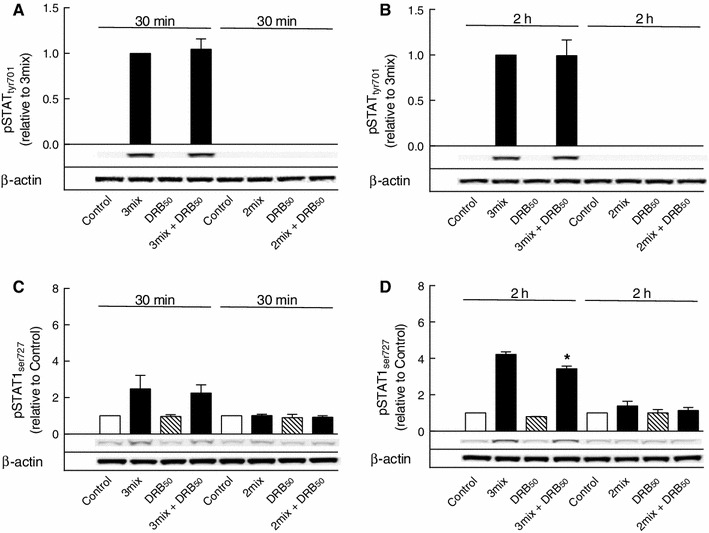
Role of IFN-γ in STAT1 phosphorylations. INS-1E cells were cultured for 30 min or 2 h in the absence or presence of IL-1β and TNF-α with IFN-γ (3mix) or without IFN-γ (2mix) and with DRB (50 μM) (DRB_50_) as indicated before determination of **a**, **b** STAT1 phosphotyrosine 701 (pSTAT_tyr701_) and **c**, **d** STAT1 phosphoserine 727 (pSTAT1_ser727_). Results are mean ± SEM (*n* = 3) with a representative experiment shown below *bars*. **d** *P < 0.01 versus 3 mix

**Fig. 11:**
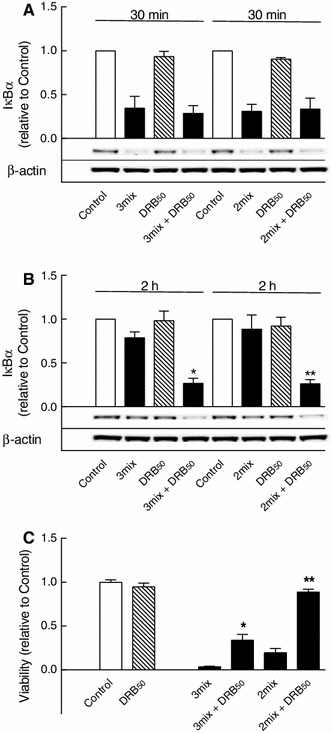
Role of STAT1 in CK2-dependent NFκB activation and β cell death. **a**, **b** INS-1E cells were cultured for 30 min or 2 h in the absence or presence of IL-1β and TNF-α with IFN-γ (3mix) or without IFN-γ (2mix) and with DRB (50 μM) (DRB_50_) as indicated before determination of IκBα. Results are mean ± SEM (*n* = 3) with a representative experiment shown below *bars*. **b** *P < 0.001 versus 3mix, **P < 0.001 versus 2 mix. **c** INS-1E cells were cultured for 48 h in the absence or presence of IL-1β, TNF-α with IFN-γ (3mix) or without IFN-γ (2mix) and with DRB (50 μM) (DRB_50_) as indicated before determination of cell viability. Results are mean ± SEM (*n* = 7). *P < 0.001 versus 3mix, **P < 0.001 versus 2mix

While IFN-γ stimulated phosphorylation of STAT1 at both tyrosine 701 and serine 727 (Fig. 10), IFN-γ, however, did not affect IL-1β and TNF-α-induced degradation and resynthesis of IκBα (Fig. 11a, b), and furthermore, DRB inhibition of IκBα resynthesis, as observed after 2 h of exposure, occurred independent of IFN-γ (Fig. 11b). Thus, although IFN-γ potentiated cytokine-induced β cell death (Fig. 11c), protection by DRB (50 μM) against cytokine-induced β cell death was not dependent on IFN-γ (Fig. 11c).

### Role of CK2 in glucose-induced insulin secretion

**Fig. 12:**
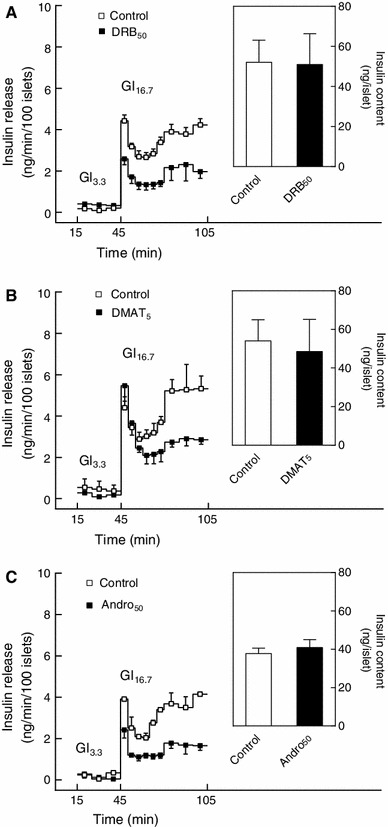
Role of CK2 and NFκB in glucose-induced insulin secretion. Mouse islets were cultured for 24 h in the absence or presence of **a** DRB (50 μM) (DRB_50_), **b** DMAT (5 μM) (DMAT_5_) or **c** andrographolide (50 μM) (Andro_50_) before determination of islet insulin secretion and insulin content as indicated. Islets were perifused for 45 min with 3.3 mM glucose (Gl_3.3_) and then stimulated for 60 min with 16.7 mM glucose (Gl_16.7_). Results are mean ± SEM (*n* = 3–4)

Since glucose-induced insulin secretion has been described to require NFκB activity [29, 30], the effects of CK2 inhibition on glucose-induced insulin secretion were evaluated. While DRB (50 μM) and DMAT (5 μM) per se did not affect the viability of mouse islets (Fig. 1), culture of mouse islets for 24 h with DRB or DMAT appeared to lower glucose-induced insulin secretion without affecting islet insulin content (Fig. 12a, b). Thus, glucose (16.7 mM)-induced insulin secretion during 1 h of perifusion was reduced to 50 ± 9 (4) % (*P* < 0.001) and to 69 ± 8 (3) % (*P* < 0.01) of controls after culture with DRB (50 μM) and DMAT (5 μM), respectively.

Likewise, culture for 24 h with the NFκB inhibitor andrographolide (50 μM), which per se did not affect the viability of mouse islets (Fig. 4), reduced glucose (16.7 mM)-induced insulin secretion to 51 ± 17 (3) % (*P* < 0.001) of controls without affecting islet insulin content (Fig. 12c).

## Discussion

According to the present experiments, CK2 may have an indispensable and specific function in cytokine-induced β cell death. Hence CK2 inhibitors failed to affect both H_2_O_2_- and palmitate-induced β cell death, but appeared to prevent IL-1β plus TNF-α-induced activation of NFκB, which is considered to be a master regulator of cytokine-induced β cell death. Indeed, while cytokines apparently failed to stimulate CK2 activity, constitutive active CK2 phosphorylated NFκB p65 at serine 529, which is a specific phosphorylation site for CK2.

In previous studies in other tissues, several sites in the NFκB pathway have been shown to be subject to phosphorylation by CK2. Thus, CK2 has been found to phosphorylate serines of IκBα, NFκB p65, and HDACs 1–3 [8–14, 17–19]. It may, therefore, seem unexpected, that CK2 inhibitors in the present study only affected the constitutive phosphorylation of NFκB p65 at serine 529. This selectivity, however, may reflect that only NFκB p65 serine 529 is under dynamic control by CK2.

Phosphorylation of NFκB p65 at serine 529 has previously been demonstrated to be indispensable in intestinal epithelial inflammation [14]. The relative importance of NFκB serine 276, 536, and 529 phosphorylations in cytokine signaling in pancreatic β cells is, however, not known, and since in one study only phosphorylation of serine 276 seemed to be of significance in cytokine signaling in β cells [31], it remains to be fully established that the sole dephosphorylation of serine 529 explains the inhibition of NFκB activity and cell death by CK2 inhibitors.

Cytokines have been described to activate p38 MAP kinase in pancreatic islets [32, 33], and p38 MAP kinase has been described to activate CK2 in other tissues [34, 35]. The role of p38 MAP kinase in cytokine-induced β-cell death is, however, disputed, and some data suggest that cytokines may not activate p38 MAP kinase in pure β-cell populations [36]. Accordingly, the p38 MAP kinase inhibitor SB203580 did not prevent cytokine-induced β cell death in INS-1E β cells in this (results not given) and a previous study [24], suggesting that CK2 does not promote β cell death through cytokine activation of p38 MAP kinase.

Cytokines have also been described to activate ERK 1/2 MAP kinase, which may fortify NFκB activation of iNOS expression [31]. Furthermore, ERK 1/2 may be activated by CK2 through phosphorylation of serines 244 and 246 [37]. In our hands, however, the ERK 1/2 MAP kinase inhibitor PD98059 did not inhibit cytokine-induced death of INS-1E β cells [24], suggesting that CK2 does not promote β cell death through cytokine activation of ERK 1/2 MAP kinase.

A few studies have suggested a possible inhibitory role for CK2 in β cell function [38, 39]. Recently, CK2 has been described to inhibit PDX-1 activity and glucose-induced insulin biosynthesis in MIN6 β cells [40, 41]. Thus, CK2 may phosphorylate PDX-1 at threonine 231 and serine 232, and culture of MIN6 β cells with CK2 inhibitors, including DMAT, has been described to increase insulin content and glucose-induced insulin secretion [40, 41]. Since PDX-1 may inhibit apoptosis in β cells [42], it, therefore, remains to be established whether CK2 inhibition of PDX-1 may promote cytokine-induced β cell death.

In similar experiments in mouse islets, however, culture with DRB (50 μM) or DMAT (5 μM) per se did not affect islet insulin content, but caused an inhibition of glucose-induced insulin secretion. The reason for this discrepancy from MIN6 β cells [40, 41] is not known, and, in fact it remains to be established whether this inhibition of glucose-induced insulin secretion also may rely on an inhibition of NFκB activity, which, as confirmed in the present study, is required for glucose-stimulated insulin secretion [29, 30]. Thus, although DRB and DMAT also may inhibit some other protein kinases apart from CK2 [43], DRB ,and DMAT, as described herein, appear to be quite specific CK2 inhibitors in pancreatic β cells.

While IL-1β and TNF-α induce NFκB activity, IFN-γ may potentiate NFκB nuclear translocation and activity through activation of STAT1 [3, 21]. Thus, IFN-γ activates JAKs 1 and 2 which stimulates STAT1 through phosphorylation of tyrosine 701 [2]. In addition, studies in other tissues have shown that phosphorylation of serine 727 by a CK2-dependent mechanism may be needed for full activation of STAT1 [20]. Although this phosphorylation is dependent on CK2, it may be exerted by a yet unknown IFN-γ-stimulated downstream protein kinase, dependent on constitutive CK2 activity [20].

According to the present experiments, IFN-γ stimulated STAT1 phosphorylation at both tyrosine 701 and serine 727 and potentiated IL-1β plus TNF-α—induced β cell death. Indeed phosphorylation of serine 727 appeared to be dependent on CK2 activity. The significance of this phosphorylation of serine 727 for STAT1 activity is, however, at present not known, and although IFN-γ potentiated cytokine-induced cell death, STAT1 was not the primary target for CK2 in cytokine-induced β cell death.

In conclusion, the present study has demonstrated that CK2 may have an indispensable function in cytokine-induced β cell death, which primarily may be exerted by NFκB activation and phosphorylation of NFκB p65 at serine 529.

## References

[1] Cerf ME (2013). Beta cell dynamics: beta cell replenishment, beta cell compensation and diabetes. Endocrine.

[2] Melloul D (2008). Role of NF-κB in β-cell death. Biochem. Soc. Trans..

[3] Cnop M, Welsh N, Jonas J-C, Jörns A, Lenzen S, Eizirik DL (2005). Mechanisms of pancreatic β-cell death in type 1 and type 2 diabetes. Many differences, few similarities. Diabetes.

[4] Donath MY, Böni-Schnetzler M, Ellingsgaard H, Halban PA, Ehses JA (2010). Cytokine production by islets in health and diabetes: cellular origin, regulation and function. Trends Endocrinol. Metab..

[5] Singh NN, Ramji DP (2008). Protein kinase CK2, an important regulator of the inflammatory response?. J. Mol. Med..

[6] Thams P, Capito K, Hedeskov CJ (1986). Polyamine-enhanced casein kinase II in mouse pancreatic islets. Diabetologia.

[7] Perkins ND (2007). Integrating cell-signalling pathways with NF-kappaB and IKK function. Nat. Rev. Mol. Cell Biol..

[8] McElhinny JA, Trushin SA, Bren GD, Chester N, Paya CV (1996). Casein kinase II phosphorylates I kappa B alpha at S-283, S-289, S-293, and T-291 and is required for its degradation. Mol. Cell. Biol..

[9] Schwarz EM, Van Antwerp D, Verm IM (1996). Constitutive phosphorylation of IkappaBalpha by casein kinase II occurs preferentially at serine 293: requirement for degradation of free IkappaBalpha. Mol. Cell. Biol..

[10] Bird TA, Schooley K, Dower SK, Hagen H, Virca GD (1997). Activation of nuclear transcription factor NF-kappaB by interleukin-1 is accompanied by casein kinase II-mediated phosphorylation of the p65 subunit. J. Biol. Chem..

[11] Kweon S-M, Wang B, Rixter D, Lim JH, Koga T, Ishinaga H, Chen L-F, Jono H, Xu H, Li J-D (2006). Synergistic activation of NF-κB by nontypeable *H. influenza* and *S. pneumonia* is mediated by CK2, IKKβ-IκaBα, and p38 MAPK. Biochem. Biophys. Res. Commun..

[12] Wang D, Baldwin AS (1998). Activation of nuclear factor-κB-dependent transcription by tumor necrosis factor-α is mediated through phosphorylation of RelA/p65 on serine 529. J. Biol. Chem..

[13] Wang D, Westerheide SD, Hanson JL, Baldwin AS (2000). Tumor necrosis factor α-induced phosphorylation of RelA/p65 on ser^529^ is controlled by casein kinase II. J. Biol. Chem..

[14] Parhar K, Morse J, Salh B (2007). The role of protein kinase CK2 in intestinal epithelial cell inflammatory signaling. Int. J. Colorectal Dis..

[15] Sisick L, Senanayake T, Veluthakai R, Woster PM, Kowluru A (2009). A novel histone deacetylase inhibitor prevents IL-1β induced metabolic dysfunction in pancreatic β-cells J. Cell. Mol. Med..

[16] Lundh M, Christensen DP, Damgaard Nielsen M, Richardson SJ, Dahllöf MS, Skovgaard T, Berthelsen J, Dinarello CA, Stevenazzi A, Mascagni P, Grunnet LG, Morgan NG, Mandrup-Poulsen T (2012). Histone deacetylases 1 and 3 but not 2 mediate cytokine-induced beta cell apoptosis in INS-1 cells and dispersed primary islets from rats and are differentially regulated in the islets of type 1 diabetic children. Diabetologia.

[17] Pflum MKH, Tong JK, Lane WS, Schreiber SL (2001). Histone deacetylase 1 phosphorylation promotes enzymatic activity and complex formation. J. Biol. Chem..

[18] Tsai S-C, Seto E (2002). Regulation of histone deacetylase 2 by protein kinase CK2. J. Biol. Chem..

[19] Zhang X, Ozawa Y, Lee H, Wen Y-D, Tan T-H, Wadzinski BE, Seto E (2005). Histone deacetylase 3 (HDAC3) activity is regulated by interaction with protein serine/threonine phosphatase 4. Genes Dev..

[20] Harvey EJ, Li N, Ramji DP (2007). Critical role for casein kinase 2 and phosphoinositide-3-kinase in interferon-γ-induced expression of monocyte chemoattractant protein-1 and other key genes implicated in atherosclerosis. Arterioscler. Thromb. Vasc. Biol..

[21] Sekine N, Ishikawa T, Okazaki T, Hayashi M, Wollheim CB, Fujita T (2000). Synergistic activation of NF-κB and inducible isoform of nitric oxide synthase induction by interferon-γ and tumor necrosis factor-α in INS-1 cells. J. Cell. Physiol..

[22] Kharroubi I, Ladriére L, Cardozo A, Dogusan Z, Cnop M, Eizirik DL (2004). Free fatty acids and cytokines induce pancreatic β-cell apoptosis by different mechanisms: role of nuclear factor-κB and endoplasmic reticulum stress. Endocrinology.

[23] Lenzen S (2008). Oxidative stress: the vulnerable β-cell. Biochem. Soc. Trans..

[24] Kiaer C, Thams P (2009). Serum albumin protects from cytokine-induced pancreatic β cell death by a phosphoinositide 3-kinase-dependent mechanism. Endocrine.

[25] Mosmann T (1983). Rapid colorimetric assay for cellular growth and survival: application to proliferation and cytotoxicity assays. J. Immunol. Methods.

[26] W.S. Rasband, ImageJ. (U.S. National Institute of Health, Bethesda, 1997–2013), http://imageJ.nih.gov/ij/. Accessed 3 September 2013

[27] Thams P, Capito K (2001). Differential mechanisms of glucose and palmitate in augmentation of insulin secretion in mouse pancreatic islets. Diabetologia.

[28] Trimble ER, Renold AE (1981). Ventral and dorsal areas of rat pancreas: islet hormone content and secretion. Am. J. Physiol..

[29] Norlin S, Ahlgren U, Edlund H (2005). Nuclear Factor-κB activity in β-cells is required for glucose-stimulated insulin secretion. Diabetes.

[30] Hammar EB, Irminger J-C, Rickenbach K, Parnaud G, Ribaux P, Bosco D, Rouiller DG, Halban PA (2005). Activation of NF-κB by extracellular matrix is involved in spreading and glucose-stimulated insulin secretion of pancreatic beta cells. J. Biol. Chem..

[31] Larsen L, Størling J, Darville M, Eizirik DL, Bonny C, Billestrup N, Mandrup-Poulsen T (2005). Extracellular signal-regulated kinase is essential for interleukin-1-induced and nuclear factor κB-mediated gene expression in insulin-producing INS-1E cells. Diabetologia.

[32] Larsen CM, Wadt KAW, Juhl LF, Andersen HU, Karlsen AE, Su MS-S, Seedorf K, Shapiro L, Dinarello CA, Mandrup-Poulsen T (1998). Interlukin-1β-induced rat pancreatic islet nitric oxide synthesis requires both the p38 and extracellular signal-regulated 1/2 mitogen-activated protein kinases. J. Biol. Chem..

[33] Saldeen J, Lee JC, Welsh N (2001). Role of p38 mitogen-activated protein kinase (p38 MAPK) in cytokine-induced rat islet cell apoptosis. Biochem. Pharmacol..

[34] Sayed M, Kim SO, Salh BS, Issinger OG, Pelech SL (2000). Stress-induced activation of protein kinase CK2 by direct interaction with p38 mitogen-activated protein kinase. J. Biol. Chem..

[35] Sayed M, Pelech S, Wong C, Marotta A, Salh B (2001). Protein kinase CK2 is involved in G2 arrest and apoptosis following spindle damage in epithelial cells. Oncogene.

[36] Pavlovic D, Mandrup-Poulsen T, Eizirik DL (2000). Activation of extracellular signal-regulated kinase (ERK) 1/2 contributes to cytokine-induced apoptosis in purified pancreatic β-cells. Eur. Cytokine Netw..

[37] Plotnikov A, Chuderland D, Karamansha Y, Livnah O, Seger R (2011). Nuclear extracellular signal-regulated kinase 1 and 2 translocation is mediated by casein kinase 2 and accelerated by autophosphorylation. Mol. Cell. Biol..

[38] Zhang S, Kim K-H (1997). Protein kinase CK2 down-regulates glucose-activated expression of the acetyl-CoA carboxylase gene. Arch. Biochem. Biophys..

[39] Donelan MJ, Morfini G, Julyan R, Sommers S, Hays L, Kajio H, Briaud I, Easom RA, Molkentin D, Brady ST, Rhodes CJ (2002). Ca^2+^-dependent dephosphorylation of kinesin heavy chain on β-granules in pancreatic β-cells. Implications for regulated β-granula transport and insulin exocytosis. J. Biol. Chem..

[40] Meng R, l-Quoballi FA, Müller I, Götz C, Thiel G, Montenarh M (2010). CK2 phosphorylation of Pdx-1 regulates its transcription factor activity. Cell. Mol. Life. Sci..

[41] Meng R, Götz C, Montenarh M (2010). The role of protein kinase CK2 in the regulation of insulin production. Biochim. Biophys. Res. Commun..

[42] Johnson JD, Bernai-Mizrachi E, Alejandro EU, Han Z, Kalynyak TB, Li H, Beith JL, Gross J, Warnock GL, Townsend RR, Permutt MA, Polonsky KS (2006). Insulin protects islets from apoptosis via Pdx1 and specific changes in the human islet proteome. Proc. Natl. Acad. Sci. U.S.A..

[43] Pagano ME, Bain J, Kazimierczuk Z, Sarno S, Ruzzene M, Di Maria G, Elliott M, Orzeszko A, Cozza G, Meggio F, Pinna LA (2008). The selectivity of inhibitors of protein kinase CK2: an update. Biochem. J..

